# The Role and Potential of Nanotechnology in Improving Solubility and Enhancing Bioavailability

**DOI:** 10.3390/pharmaceutics18040478

**Published:** 2026-04-14

**Authors:** Zsolt Hosszú, Péter Pártos, Mahyar Mahdavi, Zoltán Ujhelyi, Pálma Fehér, Ádám Haimhoffer, Ildikó Bácskay, Dóra Kósa, Ágota Pető

**Affiliations:** 1 Department of Pharmaceutical Technology, Faculty of Pharmacy, University of Debrecen, Rex Ferenc Street 1, H-4002 Debrecen, Hungary; hosszu.zsolt@pharm.unideb.hu (Z.H.); partospeter0211@gmail.com (P.P.); mmahyar91@gmail.com (M.M.); feher.palma@pharm.unideb.hu (P.F.); haimhoffer.adam@pharm.unideb.hu (Á.H.); bacskay.ildiko@pharm.unideb.hu (I.B.); 2 Doctoral School of Pharmaceutical Sciences, University of Debrecen, Nagyerdei Street 98, H-4032 Debrecen, Hungary; 3 Department of Industrial Pharmaceutical Technology, Faculty of Pharmacy, University of Debrecen, Rex Ferenc Street 1, H-4002 Debrecen, Hungary; ujhelyi.zoltan@pharm.unideb.hu; 4 Pharmaceutical Ecosystem Center, University of Debrecen, Rex Ferenc Street 1, H-4002 Debrecen, Hungary

**Keywords:** nanotechnology, nanoparticles, nanocarriers, solubility, bioavailability, poorly soluble drugs, advanced technologies

## Abstract

Nanotechnology is a rapidly emerging field in pharmaceutical sciences that has shown significant potential to enhance the bioavailability of drugs, particularly those with poor aqueous solubility. Numerous nanoscale drug delivery systems, including nanocrystals, nanosuspensions, and lipid- and polymer-based nanocarriers, have been developed to overcome pharmacokinetic limitations. Through the optimization of physicochemical properties, significant improvements in drug delivery performance can be achieved. This could potentially enable advancements in the therapeutic use of various active pharmaceutical ingredients while minimizing undesirable and potentially toxic side effects. Despite their novel advantages, the development of nanoscale carriers poses many challenges, including safety concerns, high production costs, a lack of standardization, and scalability issues. Advanced formulation technologies, such as 3D printing, artificial intelligence (AI), and machine learning (ML) approaches, can help to address these challenges as they offer opportunities to achieve uniformity and more efficient production. This review provides an overview of the critical physicochemical properties of nanoparticles and potential nanotechnological approaches to enhance solubility and bioavailability, highlighting both their advantages and limitations, and summarizes prospects for further improvement.

## 1. Introduction

Over the last few decades, nanotechnology has emerged as a promising strategy in the pharmaceutical field, largely driven by the declining number of newly discovered active pharmaceutical ingredients (APIs). This decline in pharmaceutical innovation is often associated with the high cost and risk involved in the development of new drugs [1,2]. Several factors are thought to contribute to this trend, including increasing research expenditures, declining success rates, stricter regulatory requirements, and the increasing molecular complexity of therapeutics. In addition, newly developed drug candidates often exhibit suboptimal physicochemical properties that adversely affect their pharmacokinetics, such as high molecular weight and low aqueous solubility [3,4]. Consequently, although numerous molecules are adopted in early-stage research, many of them are discarded during development because of their inadequate properties. Among these properties, bioavailability is considered one of the key determinants of possible therapeutic applicability [5]. Bioavailability is the extent and rate at which a molecule (drug or metabolite) becomes available in the systemic circulation or the site of action. It has a significant effect on the outcome of pharmacological treatment and is influenced by numerous pharmacokinetic factors, including the dissolution rate, permeability, first-pass metabolism, and solubility [6,7]. In particular, insufficient therapeutic efficacy is often the consequence of poor water solubility, as it can decrease bioavailability [8,9].

Aqueous solubility, alongside permeability, have huge impacts as limiting factors for bioavailability; together, they are considered the two most significant parameters that form the basis of the biopharmaceutical classification system (BCS), which categorizes drugs into four classes based on these parameters to predict their absorption behavior [10]. The classification is presented in Table 1.

Even though molecules possessing low solubility—classified into Classes II and IV—are generally considered undesirable during pharmaceutical development due to their depleted absorbance, a significant proportion of previously approved drugs and drug candidates in development are poorly soluble: 40% in the case of the former and 90% in the latter [11]. Low bioavailability resulting from poor water solubility presents major challenges not only in pharmaceutical development but also in clinical practice, as determining an optimal therapeutic index can be complicated and may necessitate high dosages, thereby increasing the risk of toxicity [2,12].

To overcome the difficulties caused by insufficient solubility, several approaches have been developed to enhance drug bioavailability. Among these strategies, lead optimization is a key solution which involves the modification of a previously known lead compound to improve its properties, enabling it to be used more effectively [13]. One possible optimization approach is solubility enhancement, which plays an important role in bioavailability improvement [8]. There are several methods that can be used, but one of the most innovative approaches in this context is the use of nanotechnology [14].

In general, nanotechnology typically refers to the design and application of structures ranging in size from 1 to 100 nm [15]. However, this definition originates primarily from materials science and regulatory frameworks.

In the pharmaceutical and nanomedicine fields, the term “nanocarrier” is often applied more broadly, referring to drug delivery systems with dimensions up to approximately 1000 nm. This expanded definition reflects the functional perspective adopted in drug delivery research, where colloidal systems such as lipid nanoparticles, liposomes, and solid lipid nanoparticles frequently exhibit sizes well above 100 nm while still retaining nanoscale physicochemical behaviors and biological interactions. Consequently, many lipid-based delivery systems—including SLNs and related carriers—are routinely categorized as nanosystems despite their particle sizes extending into the submicron range. Explicit clarification of this broader pharmaceutical interpretation of the nanoscale is therefore important to avoid confusion with the stricter size-based definition used in materials science [16,17,18].

In the pharmaceutical industry, the utilization of nanotechnology offers numerous advantages such as improved bioavailability and safer therapeutic application. The former can be achieved through increased solubility, enhanced permeability, and prolonged circulatory lifetime, while the latter can be accomplished by targeted delivery and controlled release. Taking these into consideration, the use of nanotechnological methods can help to enhance drug bioavailability [2,19].

The aim of this review is to comprehensively summarize the importance and potential of nanotechnology in improving solubility and bioavailability, as well as discussing its limitations and future research directions.

## 2. Nanotechnology in Drug Delivery

Over the last few decades, nanotechnology has emerged as one of the most rapidly developing fields of science, with significant applications across many areas, including medicine [20]. The utilization of nanotechnological structures for medical purposes—whether in diagnostics or therapy—is referred to as nanomedicine. Due to their numerous novel physicochemical characteristics compared to their micro- and macroscopic counterparts, nanomaterials can improve the pharmacokinetic properties of drugs (e.g., solubility, permeability) and, thus, enhance their bioavailability [21,22].

Although a wide range of physicochemical properties contribute to the advantageous features of nanoscale materials, their size, morphology, and surface properties are considered among the key factors determining pharmacokinetic behaviors. These properties collectively influence critical processes such as dissolution, cellular uptake, biodistribution, and clearance. Consequently, pharmacokinetic performance arises from the interaction of structural and surface-related characteristics, rather than from any single parameter alone.

### 2.1. The Influence of Physicochemical Properties on Pharmacokinetic Behavior

#### 2.1.1. Solubility and Dissolution Rate

Solubility is a key physicochemical characteristic that strongly influences pharmacokinetics and drug bioavailability. Particle size is an important—but not exclusive—determinant of this property. As mentioned above, nanoparticles typically range in size from 1 to 100 nm, resulting in an increased surface-to-volume ratio [23]. Enhanced dissolution of poorly water-soluble drugs is primarily explained by the Noyes–Whitney equation, which describes the dependence of dissolution rate on surface area and diffusion processes at the solid–liquid interface [24]. However, it is governed by the combined effects of particle size, morphology, and surface chemistry, which together influence pharmacokinetic performance, interactions with biological environments, and transport across biological barriers. Since drug molecules must be present in dissolved form before absorption can occur, improvements in dissolution behavior may contribute to increased bioavailability, particularly for BCS Class II and IV compounds [8,25].

#### 2.1.2. Absorption and Cellular Uptake

**Figure 1:**
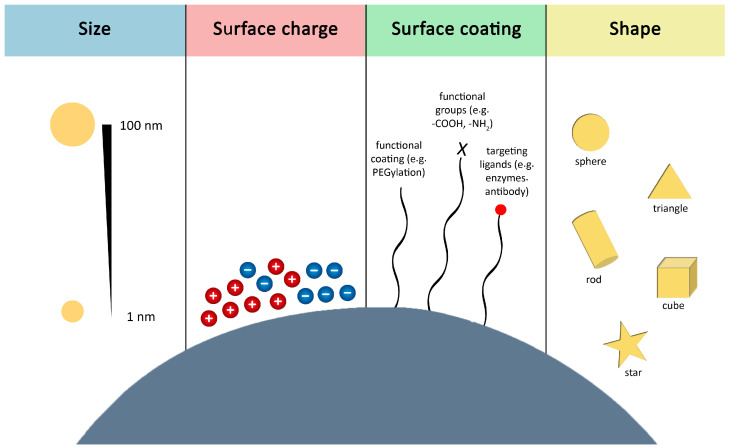
Factors influencing the cellular uptake of nanoparticles.

Nanoparticle absorption and cellular uptake occur via multiple routes, including para- and transcellular transport, and involve various passive and active internalization pathways. In general, smaller nanoparticles (1–100 nm) exhibit a higher rate of absorption and cellular uptake due to easier permeation through biological barriers and more efficient interaction with cell membranes [26,27,28,29,30,31]; however, in some cases, larger nanoparticles were internalized at a higher level [32,33]. In conclusion, a single proper size for nanoparticles cannot be defined, as their behavior is influenced by many factors. Instead, an optimal size range can be determined based on preference [34,35]. These pharmacokinetic characteristics are also defined by shape and surface properties. Different nanoparticle shapes can modify cellular interactions and uptake; for example, rods, triangles, and stars exhibit varying internalization extents even at identical sizes [36,37]. Furthermore, cellular uptake is highly affected by the surface properties of nanoparticles, including their charge and functional groups. Surface charge is a key determining factor, as it regulates the interactions with cells and the uptake mechanism. Research has shown that positively charged nanoparticles tend to be internalized to a greater extent compared to negatively charged or neutral counterparts, most likely because of the negatively charged cell membranes [38,39]. The most important factors affecting cellular uptake are summarized in Figure 1.

#### 2.1.3. Biodistribution and Blood Circulation

As nanoparticles enter the systemic circulation, their biodistribution—which is largely determined by their physicochemical properties—critically influences their therapeutic efficacy [40]. Nanoparticle size is a key determinant, as particles below a critical size threshold (~5–10 nm) may be eliminated via renal filtration [41]; however, larger nanoparticles are more likely to be taken up by phagocytic and non-phagocytic cells and even cleared by the reticuloendothelial system and organs such as the liver and spleen. Therefore, determining an optimal size range may help to avoid elimination [42]. This can be further prevented by surface modifications, including surface charge modulation and functional coatings (e.g., PEGylation), which influence the interactions of nanoparticles and circulation time [43].

For example, the presence of polyethylene glycol (PEG) on the surface of liposomes can reduce mononuclear phagocyte system uptake, thereby prolonging their circulation time. As these liposomes exhibit decreased recognition by the immune system, they are referred to as stealth liposomes [44].

#### 2.1.4. Controlled and Sustained Release

To enhance drug bioavailability, a modern approach is controlled drug release, which allows for control over the timing and rate of release, as well as the site of drug delivery [45]. Controlled and sustained drug release can be achieved via numerous methods, for instance, (a) diffusion-controlled release, (b) biodegradation-controlled release, (c) stimuli-responsive controlled release, (d) surface engineering, and (e) with the development of core–shell structures.

In diffusion- and biodegradation-controlled release systems, drug molecules are embedded within polymer matrices. In the former case, the drug diffuses through the intact polymer matrix whereas, in the latter, the biodegradation of the polymer governs the release. While diffusion-controlled systems are often associated with burst release, biodegradation-controlled systems are capable of providing sustained release [46,47,48].

Stimuli-responsive drug delivery systems are advanced strategies to control the delivery of drugs, as release only occurs when proper stimuli are applied. Numerous stimuli can be utilized to control release, such as light, temperature, pH, ultrasound, redox potential, and magnetism. These stimuli can also be combined to regulate the release tendency of these nanoparticles [49,50].

Controlled release can also be achieved via surface engineering, which involves chemical or physical modification of the nanoparticle surface. For example, conjugating specific groups on the surface of nanoparticles can control the release of drugs, and may even enhance their bioavailability [51].

Core–shell structures represent another promising strategy to achieve controlled drug release. These nanostructures are constructed as a central core enclosed by a surrounding shell. This protective shell is typically tailored to regulate the interactions between the functional core and the surrounding medium. With this structure, drug release from the core compartment can be effectively controlled, resulting in enhanced bioavailability [52].

#### 2.1.5. Targeted Delivery

One of the primary advantages of nanoparticles is their ability to facilitate targeted drug delivery, allowing the therapeutic agent to accumulate in specific sites such as tumors. This approach may augment therapeutic efficacy while attenuating toxicity in healthy tissues. Targeting strategies can be generally distinguished as passive and active. Passive targeting systems deliver nanoparticles to specific sites due to their pharmacokinetic and physicochemical properties, whereas active targeting nanoparticles are coated with specific targeting ligands that bind to receptors which are extensively expressed on the target cells [53]. As passive targeting is mostly defined by the behavior of applied nanoparticles in the circulatory system, it is highly influenced by various factors, such as size, shape, and surface properties [54]; for example, nanoparticles of different sizes may have varying efficiency in penetrating target tissues and distinct circulation times due to their differences in clearance mechanisms [55]. In active targeting, surface modifications are key determinants, as they regulate the binding affinity to specific target sites. With proper targeting ligands, drugs may be released only in tissues that express specific receptors, such as tumors, thereby improving the bioavailability and therapeutic efficacy of anticancer drugs [56].

#### 2.1.6. Reduced Systemic Toxicity and Dose Requirements

As their bioavailability is improved, a lower dose of nanoparticles is sufficient to achieve the desired therapeutic effect. This is the consequence of the above-mentioned beneficial characteristics, as an elevated fraction of the drug reaches the circulatory system and a prolonged circulation time can be achieved. This not only reduces the amount of drug that needs to be administered, but also potential side-effects and toxicity [57,58]. However, the smaller size of nanoparticles leads to increased surface reactivity, which can result in increased potential for cytotoxicity [59,60].

## 3. Nanotechnological Approaches to Enhance Solubility and Bioavailability

As discussed above, low aqueous solubility is a major limiting factor in the development of active pharmaceutical ingredients. Although various approaches have been utilized to address this challenge, nanotechnology has emerged as a particularly promising and innovative solution to enhance solubility and bioavailability. This section reviews the most extensively researched nanocarrier systems with the potential to improve suboptimal pharmacokinetic properties.

### 3.1. Nanodispersed Systems

Nanocrystals and nanosuspensions are well-dispersed colloidal systems of solid drugs, primarily designed to improve the solubility and absorption of poorly soluble drugs. Nanocrystals are defined as solid, crystalline particles with sizes usually ranging from 1 to 100 nm. Their nanoscale size endows them with unique chemical, electronic, and optical characteristics while preserving crystalline order. Nanocrystals are synthesized using various surfactants to achieve colloidal stability. In contrast, nanosuspensions are drug particles dispersed in aqueous or non-aqueous media and stabilized by surfactants and/or polymers to inhibit the aggregation of particles [61,62,63].

The application of nanocrystals and nanosuspensions has been widely investigated due to their high drug-loading capacity and versatile preparation techniques, thus potentially improving the bioavailability of poorly water-soluble drugs. These systems can enhance the dissolution rate and solubility of compounds, primarily through particle size reduction and the resulting increase in surface area. Numerous studies have demonstrated that nanocrystal formulations result in significant improvements in the aqueous solubility and dissolution behavior of BCS Class II drugs, including currently marketed pharmaceuticals and natural bioactive compounds. For instance, enhanced solubility and faster dissolution have been observed for drugs such as etoricoxib, while similar formulation approaches have been successfully applied to natural compounds with insufficient solubility, such as curcumin, leading to improved biological performance [64,65]. Nanosuspension formulations have been shown to overcome solubility-limited absorption, enhancing the bioavailability of drugs already in clinical use (e.g., efavirenz and gliclazide). However, achieving optimal pharmacokinetic performance strongly depends on the careful selection of formulation parameters [66,67].

Nanocrystals and nanosuspensions possess several benefits. They can increase the bioavailability of a drug by enhancing its solubility and their high loading capacity allows for higher concentrations, which may result in reduced dosing frequency. Although these benefits can contribute to safer therapeutic applications, limitations associated with production and stability need to be further investigated [68].

### 3.2. Lipid-Based Nanocarriers

#### 3.2.1. Liposomes

Liposomes are spherical-shaped vesicular structures with one or more lipid bilayers enclosing an aqueous core. This structure allows liposomes to carry hydrophilic and lipophilic agents, ranging from small molecules to large biomolecules. Liposomes are widely utilized to improve the bioavailability of poorly water-soluble drugs through encapsulation, owing to their nanoscale size [69]. Several studies have shown that liposomes can significantly enhance the biological performance of drugs, including poorly soluble BCS Class II and IV compounds. These carrier systems can also enable sustained release and reduce toxicity. Through proper surface modifications, including functional groups and targeting ligands, nanocarriers can achieve improved stability and enable targeted delivery, allowing predominant accumulation at specific sites, such as tumors, which can also contribute to a reduction in toxic effects [70,71,72].

The studies discussed above highlight the potential application of liposomes for enhancing solubility and bioavailability, while also providing insight into the effects of surface modifications. Liposomes have been widely investigated due to their beneficial characteristics, as they can improve efficacy and reduce toxicity via encapsulating drugs. However, due to the complexity of their production, liposomes face challenges in the pharmaceutical sector [73].

#### 3.2.2. Solid Lipid Nanoparticles (SLNs) and Nanostructured Lipid Carriers (NLCs)

Solid lipid nanoparticles (SLNs) are spherical-shaped structures ranging in size from 10 to 1000 nm. They are formed of solid lipids and can be further stabilized using surfactants. SLNs are beneficial as they are biodegradable, non-toxic, and capable of significantly improving the solubility and bioavailability of poorly water-soluble compounds. As the therapeutic efficacy of SLNs is predominantly influenced by particle size, it is important to select the most appropriate formulating lipids, which ultimately affect the rate of solubility enhancement and define the preferred administration route and release profile. Functional modifications, such as polymeric coatings, can further improve drug absorption and prolong exposures [74,75,76].

Despite their beneficial characteristics, SLNs have some limitations, such as low loading capacity, the potential for the burst release of compounds, and limited drug stability. Therefore, NLCs were designed to overcome these disadvantages. Contrary to SLNs, NLCs are composed of solid and liquid lipids, along with other excipients, to ensure stability. With properly selected excipients and physicochemical properties, NLCs can augment the pharmacokinetic behaviors (e.g., solubility, bioavailability) of drugs with typically undesirable qualities. Given the range of lipids that can form NLCs, their potential for application is extensive. Several studies have demonstrated that NLC formulations can enhance the bioavailability of various poorly soluble drugs, and additional improvements can be achieved through surface charge modifications. These systems are capable of improving the therapeutic performance of currently marketed drugs by sustaining their release, while reducing size effects [77,78,79]. Although NLCs were developed to overcome the disadvantages of SLNs, it should be noted that the excipients used in their formulations (e.g., surfactants, stabilizers, emulsifiers) may increase the risk of toxicity [80,81].

#### 3.2.3. Self-Emulsifying Drug Delivery Systems (SEDDS/SNEDDS)

Another nanotechnological approach to improve the bioavailability of poorly soluble drugs is the development of self-emulsifying drug delivery systems (SEDDS) and self-nanoemulsifying drug delivery systems (SNEDDS). These lipid-based systems offer several advantages, as their ability to self-emulsify in the gastrointestinal tract can increase their surface area for dissolution and prolong their circulation time by bypassing first-pass metabolism [82].

SNEDDS were developed to achieve nanoscale size and further advance the benefits of SEDDS, including stability and bioavailability. Several studies have validated the potential application of SNEDDS to enhance the bioavailability of numerous drugs, including those with low solubility. However, this is highly influenced by the appropriate selection of excipients, such as oils, surfactants, and co-surfactants, as different components can have varying effects on the pharmacokinetic characteristics of the formulations. Optimizing the development of SNEDDS can not only significantly enhance the dissolution rate and solubility, leading to improved pharmacokinetic profiles, but may also improve therapeutic efficacy [83,84,85].

### 3.3. Polymeric Nanocarriers

Polymeric nanoparticles are nanocarriers, typically ranging from 10 to 1000 nm in size, composed of various natural and synthetic polymers. They have been extensively utilized for drug delivery, and the physicochemical properties of these carrier systems—such as their particle size, surface charge, and polymer composition—can be carefully designed to improve solubility, stability, release kinetics, and overall bioavailability [86].

#### 3.3.1. Micelles

Polymeric micelles are self-assembled, core–shell nanoscale structures of amphiphilic molecules in aqueous media, designed to improve solubility and bioavailability. They are composed of a hydrophobic core that encapsulates the hydrophobic active ingredient, and a surrounding hydrophilic shell responsible for stabilizing the structure [87]. The proper selection of polymers is a key determinant, as they form the basis of micelles. Through optimal polymer selection, micelles can significantly improve the undesirable pharmacokinetic profiles of poorly soluble drugs, particularly their low bioavailability. Conjugating ligands to the surface of micelles can lead to further enhancement, as these ligands interact directly with specific receptors or enzymes, thereby improving therapeutic efficacy and potentially sustaining the release profile [88,89].

#### 3.3.2. Dendrimers

Dendrimers are novel nanocarrier systems designed to enhance the pharmacokinetic properties of drugs with low bioavailability. These nanoparticles are highly engineered, three-dimensional structures composed of a central unit and branching units bearing terminal functional groups. Dendrimers are considered optimal carrier systems for drug delivery, as they possess high loading capacity and can be easily customized to achieve biocompatibility and enhanced bioavailability, even for drugs with unfavorable pharmacokinetic profiles. Properly tailored dendrimers can enable sustained drug release or targeted delivery, thereby augmenting the therapeutic performance of drugs and minimizing possible toxic side effects [90,91,92].

A novel advantage of dendrimers is their potential application in gene therapy; for example, in the context of neurodegenerative disorders, Zawadzki et al. designed dendrimer–siRNA complexes that target the apolipoprotein E gene, and thus required nanocarriers capable of penetrating the blood–brain barrier. By selecting the most optimal formulation, a promising approach for gene therapy was obtained, which must be further investigated in terms of possible therapeutic applications [93].

### 3.4. Inorganic Nanocarriers

Inorganic nanocarriers are structures in the nanoscale range designed for the transport of therapeutic and imaging agents. They are composed of inorganic materials, such as metals, metal oxides, and silica-based structures, and offer benefits including high loading capacity; easily controllable size, shape, and surface properties; and the possibility of controlled release and targeted delivery [94].

**Figure 2:**
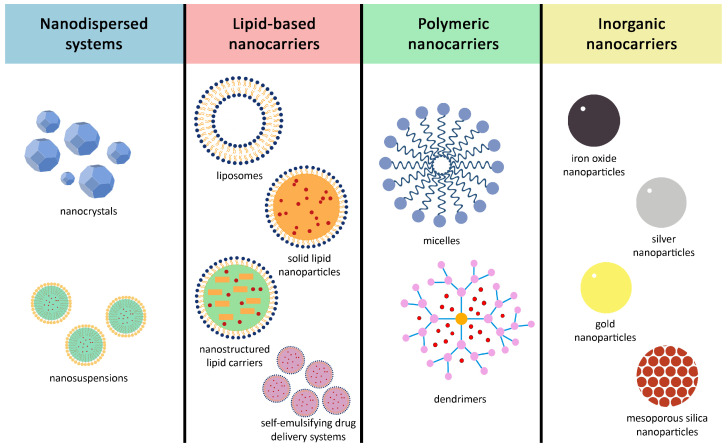
Summary of conventional nanoparticles.

Mesoporous silica nanoparticles (MSNs), as inorganic nanocarriers, have been extensively investigated due to their chemical stability, ease of synthesis, small size, large surface area, and high loading capacity. These properties allow MSNs to positively influence the solubility, bioavailability, and therapeutic effect of poorly water-soluble drugs. Further enhancements can be attained through tailored surface modifications and functionalization as these alterations can enable inorganic nanoparticles to cross the blood–brain barrier, which normally restricts the passage of drugs and reduces bioavailability [95,96]. Another advantageous property of inorganic nanocarriers is their magnetic behavior. By conjugating drugs to iron oxide nanoparticles, these inorganic nanocarriers can be actively targeted to specific sites (e.g., tumors) with the use of an active magnetic field, which may increase their bioavailability [97]. Figure 2 provides an overview of conventional nanoparticles.

## 4. Limitations of Conventional Nanotechnology Approaches

### 4.1. Issues with Stability, Aggregation, and Polymorphic Transitions

Nanoparticles are intrinsically unstable due to high surface area-to-volume ratios, which create elevated surface free energy. This drives aggregation, increasing particle size, broadening distributions, and altering pharmacokinetics. Environmental factors such as ionic strength or biomolecules can accelerate aggregation, reducing dispersion stability and delivery reliability. Lipid- and polymer-based systems can undergo polymorphic phase transitions that change their internal structure, promote drug leakage, and reduce reproducibility and performance in vivo. Limited stability undermines reliable drug delivery and reproducibility. The combination of aggregation and polymorphic instability sabotages the reproducibility, controlled release, and long-term performance of conventional nanocarriers, highlighting the need for advanced stabilization strategies [98].

### 4.2. Limited Drug Loading in Classical Carriers

Traditional nanoparticle formulations frequently have low drug loading capacity because they encapsulate drugs, rather than constituting the drug itself.

Typical loading percentages are constrained and often fall below 5–30% of total mass, demanding higher excipient volumes and reducing therapeutic efficiency [99]. Low loading capacity also increases the risk of premature drug leakage during storage or circulation, reducing effective payload delivery to target sites. Weak intermolecular interactions between the drug and carrier aggravate this effect, particularly for poorly soluble or structurally complex drugs [98].

### 4.3. Scalability and Manufacturing Challenges

Methods optimized at laboratory scale for nanoparticle synthesis (e.g., solvent evaporation, self-assembly) are difficult to translate to industrial manufacture without altering critical quality attributes. Maintaining uniform particle size, morphology, drug encapsulation, and surface properties across large batches is technically demanding and cost intensive. Scale-up processes lack standardized protocols, limiting product consistency and, thereby, commercial viability [100].

### 4.4. Regulatory Constraints and Safety Uncertainties

Nanomedicines face inadequate regulatory frameworks, as traditional drug and device regulations, were not originally designed to address the unique properties of nanoscale drug delivery systems. Many regulatory agencies, including the U.S. Food and Drug Administration (FDA) and the European Medicines Agency (EMA), still rely largely on conventional evaluation pathways, which may not fully capture the distinct pharmacokinetics, biodistribution, physicochemical behavior, surface characteristics, and multifunctionality of nanoparticle-based therapies [101,102].

Although regulatory agencies have begun to address these challenges through dedicated guidance documents, the framework remains under continuous development. For example, the FDA published the guidance document *Drug Products, Including Biological Products, that Contain Nanomaterials* (2022), which outlines considerations for the characterization, manufacturing, and quality evaluation of nanomaterial-containing drug products [103]. Similarly, the EMA has initiated several regulatory efforts addressing nanomedicines, including concept papers and guidance development initiatives focusing on the safety evaluation of nanoparticle-based medicinal products [104]. Despite these developments, the regulatory landscape for nanomedicines is still evolving, and the partial mismatch between conventional regulatory frameworks and the complex nature of nanomaterial-based therapeutics may complicate safety and efficacy assessments [101]. This can contribute to prolonged review timelines, inconsistent approval decisions, and delays in clinical translation [101,102,105].

### 4.5. Rationale for Moving Toward Advanced and Multifunctional Nanosystems

Conventional nanocarriers often deliver a single function (e.g., passive drug carriage), which limits their ability to address complex disease mechanisms and biological barriers encountered in vivo. This shortfall drives the development of advanced and multifunctional platforms that integrate multiple capabilities into one engineered system. Multifunctional and stimuli-responsive nanocarriers provide targeted delivery and controlled release, enhancing therapeutic efficacy while reducing systemic side effects by releasing drugs only at specific sites in response to endogenous (e.g., pH, enzymes) or exogenous stimuli [106]. These advanced systems improve bioavailability and intracellular co-delivery through active targeting ligands and responsive materials that change structure under defined conditions, allowing for better accumulation at disease sites and an increased local drug concentration compared with conventional carriers [107]. Multifunctional designs also allow for the co-delivery of multiple therapeutic agents or modalities (e.g., chemotherapy plus imaging or synergistic combinations), enabling spatiotemporal control over dosing ratios and sequences that are not achievable with single function carriers.

This co-delivery enhances overall therapeutic outcomes and can overcome issues such as multidrug resistance or heterogeneous tumor microenvironments by targeting multiple pathways simultaneously [108].

## 5. Advanced Formulation and Manufacturing Technologies

### 5.1. Microfluidics for Nanoformulation

#### 5.1.1. Precision Control of Particle Size and Polydispersity

Microfluidic platforms provide precision control over particle size and polydispersity by regulating flow conditions such as flow rate ratios (FRRs) and total flow rate (TFR), mixing rates, and solvent exchange in microchannels, enabling reproducible synthesis of nanoparticles with narrow size distributions. Microfluidically produced multicomponent polymeric nanoparticles are significantly smaller (24–43 nm) than bulk-prepared particles (52–65 nm) and exhibit a narrower size distribution. It has been demonstrated that increasing the FRR leads to reduced particle size and improved uniformity. It has also been shown that microfluidic mixing provides more homogeneous nanoparticle populations compared to conventional nanoprecipitation. Existing data confirm that particle characteristics can be reproducibly tuned by modifying controlled flow parameters, thus establishing microfluidics as a method that is capable of generating predictable and well-defined nanoscale systems [109].

#### 5.1.2. Continuous Manufacturing Advantages

Microfluidic nanoparticle production mainly supports continuous flow synthesis (segmented flow or droplet-based flow is the other synthesis method), which enhances reproducibility and process control compared with traditional batch operations. Continuous microreactor systems maintain defined flow conditions over time, enabling consistent generation of nanoparticles with uniform characteristics throughout production runs. Continuous flow approaches offer superior reproducibility compared to batch methods, as the reaction conditions remain constant and can be finely tuned via flow parameters. Scalable strategies such as parallelization of microfluidic channels allow for increases in throughput without degrading product quality, maintaining narrow size distributions and controlled nanoparticle properties as production scales. These features reduce batch-to-batch variability and align microfluidic production with industrial expectations for quality and consistency in nanomaterial manufacturing [110].

#### 5.1.3. Ability to Produce Hybrid and Multicomponent Nanosystems

Microfluidic platforms facilitate the production of hybrid and multicomponent nanosystems by providing environments where multiple materials can be co-assembled under controlled conditions. Research has shown that microfluidic synthesis enables the creation multilayered lipid–polymer hybrid nanoparticles by simultaneously introducing lipid and polymer streams into a microfluidic device, resulting in defined multilayer cores that combine chemical diversity and biocompatibility. These hybrid nanoparticles produced under controlled flow have potential for improved drug loading and encapsulation of poorly soluble drugs, illustrating the ability of microfluidics to integrate diverse components into a single, structurally organized nanocarrier [111].

### 5.2. Three-Dimensional Printing and Additive Manufacturing of Nano-Enabled Dosage Forms

#### 5.2.1. Implantable or Oral Dosage Forms Incorporating Nanoformulations

Three-dimensional printing enables the transformation of nanomedicine formulations into solid oral dosage forms, overcoming limitations of conventional tableting that can compromise nanoparticle stability.

Liquid nanocarrier systems such as polymeric nanoparticles, liposomes, nanoemulsions, nanosuspensions, and self-nanoemulsifying drug delivery systems (SNEDDS) can be integrated into printable feeds and then fabricated into tablets or suppositories for oral or local administration. Extrusion-based 3D printing techniques such as fused deposition modeling (FDM) and pressure-assisted microsyringe (PAM) are commonly used to build these nano-enabled solid dosage forms in a layer-by-layer manner from semisolid or filament feeds containing nanocarriers. This approach preserves the nanonization characteristics of drug-loaded carriers during printing, mitigating the loss of nano-specific properties that might occur with traditional compression methods. Three-dimensional printing also facilitates customized dose fabrication by allowing for digital geometric design before production, enabling modulation of drug content and release profiles. This technology has been proven effective for poorly soluble drugs, as using nanotechnology in drug formulations can improve solubility and dissolution behaviors relative to conventional tablets. Three-dimensional-printed solid forms containing nanocarriers could achieve controlled disintegration times while retaining a nanoscale size distribution upon release. The integration of nanomedicine and additive manufacturing thus provides a platform for next-generation oral and potentially implantable dosage forms that combine nanoscale therapeutic delivery with personalized structural design [112].

#### 5.2.2. Personalized Medicine Applications

Three-dimensional printing technologies enable dose customization and patient-specific oral dosage forms by adjusting digital design parameters, rather than changing formulation composition. For example, regarding 3D-printed tablets loaded with polymeric nanocapsules, it has been demonstrated that design variables such as infill percentage and structure influence drug release profiles, indicating potential for tailored therapy. This approach supports individualized dosing and modified release kinetics considering specific patient needs. Researchers have described how combining nanotechnology with additive manufacturing can produce customized drug delivery systems that bridge patient requirements with formulation design. Three-dimensional-printed tablets containing nanocapsules have been successfully produced, where drug loading and release were influenced by the tablet structure and printing parameters. These findings show that additive manufacturing allows for the integration of nanocarrier systems into personalized solid dosage forms [113].

#### 5.2.3. Nano-Enabled Films, Patches, and Orodispersible Systems

Emerging research has described the incorporation of nanomaterials into 3D-printed oral films and other thin printed constructs to enhance drug dissolution and delivery performance. Incorporating nanocrystals into 3D-printed oral films improved solubility and release profiles compared with non-nanocomposite printed films, demonstrating the benefits of nanoscale material inclusion in flexible dosage matrices. Nanocomposite integration has also been shown to influence mechanical properties such as flexibility and disintegration time in printed oral films, which are relevant for orodispersible and buccal applications. Furthermore, the incorporation of nanomaterials such as nanoparticles and micro-ribbons significantly altered the mechanical and flow properties of printable polymers, which is important when designing printed patch-like systems for drug delivery. These advancements demonstrate that additive manufacturing further allows nanotechnology to be merged with film and patch platforms to create nano-enabled dosage forms, thus moving beyond conventional tablets [114].

### 5.3. Artificial Intelligence and Machine Learning in Nanocarrier Design

#### 5.3.1. Predicting Solubility Enhancement Through Nanosizing

Machine learning has been used to predict nanocrystal characteristics that are closely linked to solubility enhancement, particularly particle size and polydispersity index. Models have been developed using experimental datasets from wet ball milling, high-pressure homogenization, and antisolvent precipitation. A Light Gradient Boosting Machine (LightGBM) algorithm demonstrated strong predictive performance for nanocrystal size, identifying key formulation and process parameters such as milling time and stabilizer concentration. The study demonstrates that accurate prediction of nanocrystal particle size and polydispersity can guide formulation development by highlighting critical process and formulation parameters. This approach allows for the data-driven design of nanocrystals, improving efficiency and reducing reliance on empirical trial-and-error during nanosizing optimization [115].

#### 5.3.2. Optimization of Formulation Parameters

A hybrid machine learning physics-informed modeling framework has been developed to optimize curcumin nanocomposite formulations by predicting both drug loading efficiency (LE%) and encapsulation efficiency (EE%). The study evaluated multiple ML regressors and found that a Gradient Boosting Regressor (GBR) achieved the highest predictive power for both LE% and EE% based on 74 experimentally synthesized formulations. Feature importance analysis revealed that polymer ratio and surfactant concentration were the most influential formulation variables affecting nanocomposite performance. The hybrid model enhanced generalization and reduced the need for extensive experimental screening. By identifying optimal combinations of formulation and process parameters associated with desirable performance ranges, such as particle size and zeta potential, the study demonstrated that machine learning can streamline the optimization of nanocarrier design and reduce experimental costs and time [116].

#### 5.3.3. Data-Driven Design of Hybrid Nanocarriers and Smart Nanosystems

A recent review on machine learning in nanomedicine has described how ML algorithms can be applied to predict critical quality attributes and biological performance of diverse nanoparticulate drug delivery systems, such as polymeric nanoparticles, lipid-based carriers, and hybrid nanostructures. ML models have been used to analyze large datasets covering formulation variables and performance outcomes to identify patterns and relationships that traditional trial-and-error methods might miss. These models are capable of predicting attributes such as particle size, shape, surface properties, drug encapsulation efficiency, and release behavior based on input features derived from experimental data. By leveraging ML for predictive modeling, researchers can guide the rational design of multifunctional and hybrid nanocarrier systems that balance stability, encapsulation, and delivery performance. Machine learning offers powerful data-driven tools to accelerate formulation design and the optimization of complex nanocarriers beyond conventional empirical workflows [117].

### 5.4. Supercritical Fluid Technology

#### 5.4.1. Supercritical CO_2_-Based Nanonization

Supercritical carbon dioxide (scCO_2_)-based technologies enable the formation of drug nanoparticles and nanocrystals by exploiting the unique solvent and antisolvent properties of scCO_2_ under controlled pressure and temperature. The particle size and morphology can be tuned by adjusting operational variables such as pressure, temperature, and solvent, allowing precise control over the size distribution. These scCO_2_-based nanonization methods offer advantages over traditional micronization techniques by producing smaller, more uniform particles with fewer residual solvents.

In their comprehensive review, Padrela et al. emphasized the versatility of scCO_2_ in generating amorphous/crystalline pharmaceuticals at the nanoscale with better reproducibility [118].

#### 5.4.2. Production of Solvent-Free, High-Purity Nanoparticles

Supercritical fluid extraction of emulsions (SFEE) uses scCO_2_ to rapidly remove organic solvent from emulsions, leading to uniform micro- and nanoparticles with high purity and significantly reduced residual solvent compared with conventional solvent evaporation or spray-drying. The fast mass transfer and high solvent power of scCO_2_ during SFEE improve drug encapsulation efficiency, particle size control, and prevention of particle agglomeration. Because scCO_2_ leaves essentially no organic solvent behind upon depressurization, the resulting nanoparticles have very low solvent residues, meeting quality requirements for pharmaceutical applications. SFEE has the ability to produce small, spherical, and monodisperse particles across a range of drug types [119].

#### 5.4.3. Suitability for Thermolabile APIs

Supercritical CO_2_ has a critical temperature of 31.1 °C, enabling processing at temperatures significantly lower than many conventional drying or melting techniques. Supercritical antisolvent (SAS) and related methods are particularly advantageous for temperature-sensitive compounds, as particle precipitation occurs without exposure to high heat. Successful processing of thermolabile drugs can be achieved while maintaining chemical stability, demonstrating that supercritical fluid technology is well suited for APIs that may degrade under conventional thermal manufacturing conditions [120].

## 6. Safety, Regulatory, and Translational Considerations for Next-Generation Nanosystems

### 6.1. Unique Toxicity Concerns of Newer Hybrid and Biomimetic Systems

Hybrid and biomimetic nanosystems combine synthetic materials with biological components, generating complex toxicity profiles that conventional nanocarriers do not exhibit. Their small size and large surface area enable their widespread distribution in tissues, exposing multiple organs to potential toxic effects. Their surface chemistry, particle shape, and incorporation of functional elements such as targeting ligands or polymers can affect whether oxidative stress, cytotoxicity, and inflammatory responses are triggered.

A key factor influencing the biological response to nanoparticles is the formation of the so-called protein corona. When nanoparticles enter biological environments such as blood or interstitial fluids, proteins and other biomolecules rapidly adsorb onto their sur-face, forming a dynamic biomolecular layer that effectively defines the nanoparticle’s bio-logical identity rather than its pristine synthetic surface [121]. This biomolecular coating can significantly alter physicochemical properties such as surface charge, colloidal stability, and cellular uptake, thereby influencing their biodistribution, immune recognition, and clearance pathways [121,122]. Consequently, protein corona formation may promote immune activation, complement responses, or hypersensitivity reactions, depending on the composition of the adsorbed biomolecules and the physicochemical properties of the nanoparticle surface [122].

In addition, metal-based or polymeric components may release ions or degradation products that accumulate over time, amplifying the risk of toxicity [123]. Dose-dependent and cumulative effects make long-term exposure studies essential, particularly for repeated or chronic administration scenarios. Standard in vitro assays often fail to capture multi-organ interactions, and animal models may not fully replicate human physiology, complicating toxicity predictions.

Comprehensive preclinical evaluation, combining both in vitro and in vivo approaches, is necessary to assess organ-specific toxicity, systemic effects, and safe dosing ranges. Emerging studies have also emphasized the need for mechanistic understanding of hybrid and biomimetic interactions with immune and metabolic systems, in order to guide rational design and regulatory approval [122,124].

### 6.2. In Vivo Fate and Long-Term Biocompatibility Challenges

Advanced nanosystems display complex and often unpredictable biological fates, as their physicochemical properties govern interactions with plasma proteins, immune cells, and physiological barriers, which ultimately determine their biodistribution and clearance. In multiple in vivo studies, nanoparticles such as BSA-coated gold particles were found to persist in major organs long after administration. In particular, significant amounts remained in the liver, spleen, and kidneys up to 120 days post-injection, and prolonged retention is associated with inflammatory and fibrotic responses, indicating that slow elimination can induce chronic biological effects despite initial biocompatibility assumptions [125]. Biodistribution reviews have shown that many nanomaterials accumulate in the reticuloendothelial system (RES) because clearance by phagocytic cells traps particles in filtering organs, with mechanisms governing subsequent translocation and cellular processing remaining poorly understood. Persistent sequestration can lead to prolonged tissue exposure, and biological transformations such as protein corona formation and intracellular degradation change the particle identity over time, affecting cellular uptake, immune recognition, and organ-specific responses. These dynamic in vivo processes are not well captured by acute toxicity tests or in vitro models, which cannot replicate whole-organ system interactions or long-term kinetic behavior, making it difficult to predict chronic safety profiles [126].

### 6.3. Analytical Challenges for Complex, Multicomponent Nanocarriers

Complex, multicomponent nanocarriers present analytical challenges in accurately characterizing size and surface modifications, because standard methods are often insufficient for advanced formulations. Measuring size across a wide range of nanoparticle types, evaluating polydisperse systems, and analyzing surface modifications remain difficult with currently available tools. Techniques such as dynamic light scattering (DLS) and electron microscopy provide important data, but have limitations in resolving heterogeneity, non-spherical shapes, and surface functionalization in hybrid systems. Moreover, there is a lack of validated reference materials and standardized protocols for the characterization of complex surface chemistries, which makes comparison across studies and regulatory assessments inconsistent. While there exist some methods to analyze surface modifications, their feasibility and accuracy when applied to real, multicomponent nanoparticles are still uncertain, leaving significant gaps in analytical capability. This analytical gap impedes the establishment of critical quality attributes and hampers reproducibility and regulatory evaluation of advanced nanomedicine platforms [127]. Surface functionalization complicates nanoparticle characterization because biological interfaces dynamically alter particle surfaces. The formation of a protein corona in biological fluids changes the apparent surface chemistry, charge, and targeting functionality of nanoparticles, which can affect cellular interactions. Traditional isolation methods, such as centrifugation or filtration, may remove loosely bound proteins and disrupt the native corona, leading to inaccurate representation of in vivo particle identity [128].

### 6.4. Regulatory Gaps and Unmet Needs in Standardization

Regulatory frameworks for nanomedicines remain underdeveloped and inconsistent, largely because existing pharmaceutical regulations were designed for conventional small molecules or biologics, not complex nanoparticle-based products.

Agencies worldwide lack harmonized definitions, test criteria, and classification standards that capture the unique properties of nanomedicines, such as their size-dependent behaviors and multifunctionality, leading to divergent regulatory pathways and approval requirements across regions [129]. The absence of clearly defined standardized characterization methods and reference materials hampers reliable quality control and method validation. Regulators and developers cannot consistently compare critical quality attributes across studies or batches without agreed protocols, which slows evaluation and introduces uncertainty into safety and efficacy assessments [130]. Standard bioequivalence paradigms also fall short for nanomedicines and their follow-on versions. The classical generic paradigm based on plasma drug concentrations does not necessarily reflect equivalence of nanoparticle behavior, making regulatory assessment of nanosimilars particularly challenging [131].

### 6.5. Considerations for Industrial Scale-Up

Industrial scale-up of nanoparticle manufacturing is constrained by the challenge of maintaining critical quality attributes during process scaling. Methods such as high-pressure homogenization, solvent evaporation, and microfluidic synthesis are optimized at small volumes, where mixing, mass transfer, and heat transfer are precisely controlled. These conditions change unpredictably when scaled to industrial reactors, leading to shifts in particle size distribution, morphology, and surface properties. Researchers have explicitly noted that reproducibility and consistency become major barriers as scale increases, because nanoscale assembly is highly sensitive to process parameters that are difficult to control uniformly at larger volumes. Maintaining uniform physicochemical properties across batches is central to therapeutic performance, yet batch-to-batch variability remains a persistent issue in clinical translation. Moreover, comprehensive in-process monitoring and quality control become more complex at scale, requiring sophisticated analytical tools and real-time process insights that are still under development. Due to these factors, strategies for scalable, robust production must integrate advanced process design, rigorous parameter control, and quality management frameworks to ensure that industrial output matches laboratory performance [132]. Specific materials, such as PLGA-based nanoparticles, illustrate these barriers; in particular, industrial scale-up requires transferring small-scale synthesis protocols into scalable processes that ensure consistent particle assembly, encapsulation, and purity, which remains a persistent bottleneck in clinical translation [133]. Cost considerations further complicate technical challenges. Maintaining strict control over manufacturing parameters and ensuring reproducibility across batches increases production expenses relative to traditional pharmaceuticals. As a result, the commercial viability and healthcare affordability of nanomedicine products depend on developing scalable, cost-efficient production technologies that maintain quality without prohibitive cost increases [134]. Lipid nanoparticles (LNPs) enable safe, efficient mRNA delivery by protecting it from degradation and facilitating cellular uptake. The global deployment of LNP–mRNA COVID-19 vaccines demonstrated successful industrial-scale, GMP-compliant production and clinical translation, proving that complex nanocarriers can be manufactured and used effectively at scale [135].

## 7. Conclusions

In summary, nanotechnology has profoundly reshaped the field of drug delivery by overcoming the most enduring obstacles in pharmaceutical science: inadequate solubility and limited bioavailability. By precisely engineering their particle size, structural architecture, and surface functionality, nanoscale systems can provide fine-tuned control over absorption, biodistribution, circulation kinetics, and cellular internalization.

Diverse nanocarrier platforms—including lipid-based formulations, polymeric nanoparticles, dendritic systems, inorganic matrices, and hybrid assemblies—have consistently shown the capacity to improve therapeutic performance while minimizing off-target toxicity. Nevertheless, traditional nanosystems remain constrained by issues such as physicochemical instability, restricted drug loading capacity, manufacturing scale-up difficulties, analytical complexity, and evolving regulatory expectations. These constraints have stimulated the development of next-generation, multifunctional, and stimuli-responsive nanoplatforms that unify targeted delivery, controlled release, and combination therapy within integrated architectures. Concurrently, innovative production technologies—including microfluidic processing, additive manufacturing, AI-driven formulation modeling, and supercritical fluid techniques—offer transformative opportunities to enhance process robustness, scalability, and formulation precision. Despite these advances, the path to clinical translation demands comprehensive evaluation of long-term biodistribution, chronic toxicity of hybrid constructs, dynamic protein corona formation, and stringent large-scale quality assurance. The establishment of harmonized regulatory standards and validated characterization frameworks is expected to play a critical role in translating laboratory breakthroughs into clinically approved therapies. Ultimately, the integration of advanced material engineering, intelligent manufacturing strategies, and data-centric optimization heralds a new era in which nanosystems serve as foundational components of precision and patient-tailored pharmacotherapy.

## Figures and Tables

**Table 1:** BCS classification.

Class	Solubility	Permeability	Significance
I	High	High	Well absorbed
II	Low	High	Solubility limited
III	High	Low	Permeability limited
IV	Low	Low	Poorly absorbed

## Data Availability

No new data were created or analyzed in this study.

## Abbreviations

The following abbreviations are used in this manuscript:

FDA	Food and Drug Administration
SLNs	Solid Lipid Nanoparticles
NLCs	Nanostructured Lipid Carriers
HIV	Human Immunodeficiency Virus
AIDS	Acquired Immunodeficiency Syndrome
PEG	Polyethylene Glycol
SiRNA	Small interfering Ribonucleic Acid
MSNs	Mesoporous Silica Nanoparticles
BCS	Biopharmaceutical Classification System
EMA	European Medicines Agency
FRRs	Flow Rate Ratios
TFR	Total Flow Rate
SNEDDS	Self-Nanoemulsifying Drug Delivery Systems
SEDDS	Self-Emulsifying Drug Delivery Systems
FDM	Fused Deposition Modeling
PAM	Pressure-Assisted Microsyringe
LightGBM	Light Gradient Boosting Machine
LE%	Loading Efficiency
EE%	Encapsulation Efficiency
GBR	Gradient Boosting Regressor
ML	Machine Learning
scCO_2_	Supercritical Carbon Dioxide
SFEE	Supercritical Fluid extraction of emulsions
API	Active Pharmaceutical Ingredient
BSA	Bovine Serum Albumin
DLS	Dynamic Light Scattering
PLGA	Poly Lactic-co-Glycolic Acid
CO_2_	Carbon Dioxide
LPNs	Lipid Nanoparticles
mRNA	Messenger Ribonucleic Acid
COVID-19	Coronavirus Disease 2019
